# Editorial: The Role of ncRNAs in Solid Tumors Prognosis: From Laboratory to Clinical Utility

**DOI:** 10.3389/fonc.2020.631316

**Published:** 2021-02-02

**Authors:** Jian-Guo Zhou, Zhaohui Huang, Ondrej Slaby, Alfons Navarro

**Affiliations:** ^1^Department of Oncology, The Second Affiliated Hospital of Zunyi Medical University, Zunyi, China; ^2^Department of Radiation Oncology, Universitätsklinikum Erlangen, Erlangen, Germany; ^3^Comprehensive Cancer Center Erlangen-Europäische Metropolregion Nürnberg (ENM), Erlangen, Germany; ^4^Wuxi Cancer Institute, Affiliated Hospital of Jiangnan University, Wuxi, China; ^5^Laboratory of Cancer Epigenetics, Wuxi School of Medicine, Jiangnan University, Wuxi, China; ^6^Department of Pathology, University Hospital Brno, Brno, Czechia; ^7^Central European Institute of Technology (CEITEC), Masaryk University, Brno, Czechia; ^8^Molecular Oncology and Embryology Laboratory, Human Anatomy Unit, Faculty of Medicine and Health Sciences, University of Barcelona, August Pi i Sunyer Biomedical Research Institute (IDIBAPS), Barcelona, Spain; ^9^Thoracic Oncology Unit, Hospital Clinic, Barcelona, Spain

**Keywords:** long non-coding RNA, microRNA, circular RNA, the cancer genome atlas (TCGA), gene expression omnibus (GEO)

## Editorial

Today we know that non-coding RNAs (ncRNAs) represent most of the transcribed human genome and participate in relevant cellular processes. NcRNAs regulate from RNA transcription to protein translation, have important epigenetic roles or facilitate protein–protein interactions among other functions. In consequence, their dysregulation has been associated with tumor development and progression. Recently, their expression has also been detected in body fluids, opening the use of circulating ncRNAs for diagnosis and for evaluation and monitoring cancer prognosis.

The present Research Topic titled “The Role of ncRNAs in Solid Tumors’ Prognosis: From Laboratory to Clinical Utility” features 48 scientific studies (originals, meta-analysis and reviews) that display different functions that ncRNAs can make in the carcinogenesis process and how they can affect cancer treatment response and therefore, be of utility as cancer biomarkers in solid tumors. This topic includes two groups of papers: 1) articles describing mechanisms by which ncRNAs regulate cancer development and progression; and 2) articles that use both publicly available data (TCGA or GEO) or own researchers’ collected datasets to identify and validate ncRNA prognostic signatures for several cancers.

NcRNAs can be classified in two major groups according to their size; small ncRNAs and long ncRNAs (lncRNAs); the last include a very heterogeneous group that have in common that they are longer than 200 nucleotides. From the small ncRNA group, the best studied class is microRNAs (miRNAs). miRNAs are short single-stranded RNAs, transcribed as long precursors finally processed to an 18–22 nucleotide product. Although a lot of different functions have been described for miRNAs, the most relevant one is the participation in the translation process, where they inhibit in a sequence-dependent manner the mRNA translation to protein of their target mRNAs. Furthermore, their utility as cancer biomarkers in solid tumors has been extensively studied in both tumor and liquid biopsy. Several authors have addressed this issue in this Research Topic. In the next paragraphs we are summarizing the main achieved results.

Prof. Zhao and colleagues reviewed the contribution of miR-27a to solid tumor diagnosis and prognosis and discussed the possibilities to use this miRNA for therapy and drug design (Zhang J. et al.). Another review article by Xiang et al. commented on the double function (tumor suppressor or oncogene) of miR-186. They explained that while in most of the tumors miR-186 is a tumor suppressor miRNA, in endometrial and cutaneous squamous cell carcinomas it acts as an oncogene. It was discussed in the review article that a dose dependent-effect on both miRNA and its target abundance may explicate the opposing role of miR-186 depending on the tumor type. In an original study, Liu C. et al. used a different bioinformatic approach integrating miRNA targets and phenotypes to identify part of the miR-622 targetome. *In silico* data was used to study in breast cancer *in vitro* models (viability, migration, *etc*.) the role of miR-622. In another study, Di et al., using TCGA and GEO, identified a 9-miRNA signature including miR-29c, -92a, -101, -148a, -200a, -210, -338, -424 and -492, with diagnostic and prognostic utility for patients with colorectal cancer (CRC). They validated the signature and specifically studied the involvement in the metastatic process of miR-200a-3p using different *in vitro* experiments.

In relation to miRNAs involved in treatment response, Cheng and Shen identified miR-335 (downregulated) as a factor involved in radiotherapy resistance in melanoma through regulation of *ROCK1*. Lin et al. observed that miR-199b-5p was involved in angiogenesis regulation (antiangiogenic factor) in breast cancer by targeting ALK1 and suggested that this miRNA could be used as antiangiogenic treatment response biomarker. Gao et al.’s results indicated that by regulation of *TRIM27* and related PI3K/Akt pathway, this miRNA modulates *in vitro* aggressiveness of hepatocellular carcinoma (HCC). Prof. Fan, using *in vivo* and *in vitro* experiments, found that miR-24-3p was involved in pancreatic ductal adenocarcinoma (PDAC) progression through *LAMB3* downregulation (Huang et al.). Similarly, but in breast cancer, Prof. Wei revealed that miR-15b-5p regulating heparanase-2 (*HPSE2*) could be also a progression biomarker (Wu B. et al.). Prof. Lu led a research that associated serum miR-222-3p with pathological complete response, survival, and cardiotoxicity in HER2-positive breast cancer patients receiving trastuzumab-based neoadjuvant treatment (Zhang S. et al.). Prof. Edwards and his team, combining profiling and functional studies, identified key miRNAs for prostate cancer metastasis (Rao et al.). All these studies reinforced the significance and clinical utility of miRNAs in cancer.

The other articles sent to our Research Topic, specifically 29 articles, focused on the study of lncRNAs. LncRNAs are a heterogeneous class of ncRNAs since they included all ncRNAs longer than 200 nt (Gong and Jang). We have summarized the principal results by tumor type in the following lines.

Several authors have studied lncRNAs in non-small cell lung cancer (NSCLC), and different lncRNAs have been identified. TGF*β*-induced lncRNA TBULC enhanced migration and invasion *in vitro*, and its higher levels were associated with shorter overall survival (OS) in patient samples (Zheng S. et al.). LincRNA00494 repressed proliferation by regulation of the miR-150-3p/SRCIN1 axis (Dong J. et al.).

In CRC, a combination of AP003555.2, AP006284.1, and LINC01602 was identified and validated for predicting OS (Liu Y. et al.). MIR4435-2HG was also related to OS and *in vitro* studies showed that the oncogenic role of this lncRNA was performed trough miR-206 sponging and, therefore by modulation of YAP1 protein levels (Dong et al.). Another lincRNA with oncogenic potential in CRC was linc00662 which performed a ceRNA function on miR-497-5p/*AVL9* axis (Wang H. et al.). In a similar way, MIR570MG, by controlling miR-145 levels and consequently modulating the SMAD3 pathway, plays a role in regorafenib treatment resistance (Wei et al.). In contrast to the previous ones, *MEG3* lncRNA was acting as tumor suppressor in CRC where its low levels were associated with shorter OS. MEG3 lncRNA expression was affected by vitamin D and their function was related to glycolysis in CRC (Zuo et al.).

In breast cancer, LINC00993 was identified by *in vitro* and *in vivo* studies as a tumor suppressor gene in which low levels were related to shorter OS in tri-negative breast cancer patients (Guo et al.). In contrast, MRPS30-DT lncRNA acted as oncogene by regulating Jab1/Cops5, and its high levels were associated with worse outcome (Wu B. et al.). Another oncogenic lncRNA was Linc00668 which promoted a more malignant phenotype in breast cancer cells through regulation of SND1. Moreover, their higher levels were related with treatment resistance in cell lines and with shorter disease-free survival in patient samples (Qian et al.). Lastly, DCST1-AS1 enhanced epithelial–mesenchymal transition by *ANXA1* regulation and promoted *in vitro* treatment resistance in metastatic breast cancer cells (Tang et al.). Also in breast cancer, but in the context of the study of the antitumorigenic role of Huaier, a traditional Chinese medicine (see NCI drug dictionary for more detailed information, https://www.cancer.gov/publications/dictionaries/cancer-drug/def/huaier-extract-granule), Wang W. et al. identified a Huaier-related lncRNA, linc00339, which acting through the miR-4656/CSNK2B axis, participates in the antiproliferative effect of this drug in breast cancer cells.

In a more diverse set of tumor types, several authors identified interesting lncRNAs with potential use as diagnostic or prognostic markers. PICSAR, LINC00319, THOR, AK144841, MALAT1, LINC10148, and HOTAIR were found upregulated, while TINCR, LINC00520, and GAS5 were downregulated in cutaneous squamous cell carcinoma and correlated with several histological subtypes (Wang Y. et al.). In esophageal squamous cell carcinoma a 6-lncRNA signature associated with metabolic syndrome including AC005091.1, SNHG6, AC091544.4, DNAJB5-DT, HTT-AS, and ANKRD10-IT1, impacted prognosis in these patients (Liu Y. et al.). Another prognostic signature was identified also for HCC by Li W. et al., who performed *in vitro* studies with one of the lncRNAs of the signature, GACAT3, to partially validate the functional relevance of the identified signature. In PDAC, Zhou C. et al. identified and validated in two cohorts a signature of five lncRNAs: RP11-159F24.5, RP11-744N12.2, RP11-388M20.1, RP11-356C4.5, and CTC-459F4.9 as independent prognostic markers for OS. In nephroblastoma, Wang J. et al. identified lncRNAs with prognostic significance by the use of multiomic integration data, and in a similar way, but in gastric cancer Pan H. et al. identified networks of lncRNAs/miRNAs/mRNAs involved in the tumorigenesis process and focused in the *in vitro* validation of the *ADAMTS9-AS2*/miR-372/*CADM2 axis*.

Interestingly, most of the previously commented lcnRNA studies are related to the ceRNA function of lncRNAs, and they took advantage of public available databases, such as TCGA or GEO, to connect the experimental findings with the clinical utility of the potential lncRNA biomarkers identified.

Several authors have implemented review or meta-analysis studies to emphasize the relevant role of lncRNA in cancer. Lu et al. focused on the revision of lncRNA involved in metabolic cancer-related processes. Zhou Y. et al. described the most relevant lncRNAs associated with cancer-immunology, specifically PD-1/PD-L1 and CTLA-4 pathways, and their relevance for immunotherapy resistance. In the same context, Luo et al. emphasized the role of lncRNAs in the modulation of the immunosuppressive microenvironment and discussed the potential clinical applications of these lncRNAs in cancer treatment by immunotherapy. Peng et al. emphasized the most relevant lncRNAs related to thyroid cancer and their potential interactions with mRNAs and miRNAs. Finally, three papers performed a meta-analysis and a review to highlight the relevance of three lncRNAs in cancer patients’ survival; one focused on SOX2-OT lncRNA (Li Y. et al.), another on PANDAR lncRNA (Han et al.), and the last one focused on DANCR lncRNA (Jin et al.). The three studies highlighted that the overexpression of the three individual lncRNAs was related to advanced disease and worst patient outcome in several cancers.

Four articles focused on small nucleolar RNA (snRNA) host genes (SNHGs), a class of lncRNAs derived from the non-processing of the RNA sequences of the genes coding for snRNAs. On one hand, one article from Zimta et al. reviewed the oncogenic potential of this gene family in various cancers and highlighted their potential as viable biomarkers. On the other hand, three articles focused on the study of the oncogenic role of specific SNHGs: 1) SNHG17 was linked to carcinogenesis in prostate cancer through sponging miR-144, which regulates CD51 (Bai et al.); 2) SNHG12 modulated MDM4 and p53 pathways by acting on miR-129-5p in renal cell carcinoma (Wu Z. et al.); and 3) SNHG18 in gliomas was linked to *ENO1* (Zheng R. et al.). In summary, we can state that the four studies confirmed the relevance of SNHGs, which knockdown might be considered as a new cancer therapeutic option that deserves further investigation.

A more atypical group of lncRNAs are circular RNAs (circRNAs), which are characterized by the formation of a functional circular structure, generally as a result of a backsplicing process, without the 5′ cap or 3′ poly A tail (Liu J. et al.). Their implication in the tumorigenesis process has been recently confirmed, and their potential as cancer biomarkers has been understudied to date. Some articles included in this Research Topic have been dedicated to the study of circRNAs in different tumors. Wan et al. emphasized the role of circRNAs in osteosarcoma. Wang X. et al. identified and validated in HCC, hsa_circ_0000517 as a prognostic marker by bioinformatics, qRT-PCR, and Sanger sequencing. In CRC, comparing the exosomal cargo pre- and post-surgery, Pan B. et al. observed that hsa-circ-0004771 levels become downregulated after surgery, indicating a potential role for this exosomal circRNA on diagnosis and on disease follow-up. In another study, Yu et al. performed a profile of circRNAs in pheochromocytomas and paragangliomas and observed that the ceRNA function performed by the identified circRNAs was related with the pathogenesis process by regulation of the epigenetics processes mediated by histone methylation. Finally, in neuroblastoma, the *in vitro* and *in vivo* study of Yang et al. identified the circDGKB as an oncogene involved in disease progression by regulation of miR-873 and its target GLI1.

Despite the sharp increase in lncRNA-related publications (Dai et al.), including the ones from the present Research Topic, deeper functional and structural studies are still needed to understand the principles of the emerging role of lncRNAs, which is a heterogeneous group with a great potential in cancer prognosis and therapeutics. Therefore, we still need to investigate more and more novel ncRNAs as biomarkers or targets for cancer treatment and control. Let’s go ahead with translational researches of ncRNAs for cancer!

## Author Contributions

All authors listed have made a substantial, direct, and intellectual contribution to the work and approved it for publication.

## Funding

This work is supported by National Natural Science Foundation of China (NSFC) (Grant No. 82060475), Research Programs of Science and Technology Commission Foundation of Zunyi City (Grant Nos. HZ2019-11 and HZ2019-07), Research Programs of Health Commission Foundation of Guizhou Province (Grant Nos. gzwjkj2019-1-073 and gzwjkj2019-1-172) and by Ministry of Economy, Industry, and Competition, Agencia Estatal de Investigación co-financed with the European Union FEDER funds SAF2017-88606-P (AEI/FEDER, UE).

## Conflict of Interest

The authors declare that the research was conducted in the absence of any commercial or financial relationships that could be construed as a potential conflict of interest.

